# Genetic diversity of *Trypanosoma cruzi* infecting raccoons (*Procyon lotor*) in 2 metropolitan areas of southern Louisiana: implications for parasite transmission networks

**DOI:** 10.1017/S0031182023000070

**Published:** 2023-04

**Authors:** Alicia Majeau, Erin Cloherty, A. Nikki Anderson, Susanne C. Straif-Bourgeois, Eric Dumonteil, Claudia Herrera

**Affiliations:** 1 Tulane University School of Public Health & Tropical Medicine, New Orleans, LA, USA; 2 New Orleans Mosquito, Rodent, and Termite Control, New Orleans, LA, USA; 3 Louisiana Department of Wildlife and Fisheries, Baton Rouge, LA, USA; 4 Louisiana Health Sciences Center, School of Public Health, New Orleans, LA, USA

**Keywords:** Diversity, mini-exon, protozoa, raccoons, *Trypanosoma cruzi*, zoonosis

## Abstract

*Trypanosoma cruzi*, the aetiological agent of Chagas disease, exists as an anthropozoonosis in Louisiana. Raccoons are an important reservoir, as they demonstrate high prevalence and maintain high parasitaemia longer than other mammals. Given the complex nature of parasite transmission networks and importance of raccoons as reservoirs that move between sylvatic and domestic environments, detailing the genetic diversity of *T. cruzi* in raccoons is crucial to assess risk to human health. Using a next-generation sequencing approach targeting the mini-exon, parasite diversity was assessed in 2 metropolitan areas of Louisiana. Sequences were analysed along with those previously identified in other mammals and vectors to determine if any association exists between ecoregion and parasite diversity. Parasites were identified from discrete typing units (DTUs) TcI, TcII, TcIV, TcV and TcVI. DTUs TcII, TcV and TcVI are previously unreported in raccoons in the United States (US). TcI was the most abundant DTU, comprising nearly 80% of all sequences. All but 1 raccoon harboured multiple haplotypes, some demonstrating mixed infections of different DTUs. Furthermore, there is significant association between DTU distribution and level III ecoregion in Louisiana. Finally, while certain sequences were distributed across multiple tissues, others appeared to have tissue-specific tropism. Taken together, these findings indicate that ongoing surveillance of *T. cruzi* in the US should be undertaken across ecoregions to fully assess risk to human health. Given potential connections between parasite diversity and clinical outcomes, deep sequencing technologies are crucial and interventions targeting raccoons may prove useful in mitigating human health risk.

## Graphical abstract

**Figure:**
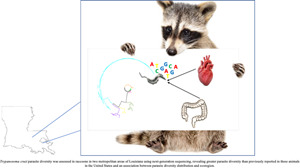

## Introduction

In southern Louisiana, and across the southern United States (US), *Trypanosoma cruzi* infection exists as an important anthropozoonosis, prevalent in both wildlife and domestically maintained species in this region. Infection has been reported in rodents, dogs, cats, non-human primates and even a domestic llama in the state (Pronovost *et al*., 2018; Elmayan *et al*., 2019; Herrera *et al*., 2019*a*; Dumonteil *et al*., 2021; Thompson *et al*., 2021). Transmission occurs through contact with infected feces of *Triatoma sanguisuga* insects, the primary vector in the state, though both oral transmission resulting from ingestion of infected insects and congenital transmission have also been suggested to contribute to wildlife infection (Roellig *et al*., 2009*b*; Kribs-Zaleta, 2010). Though infrequent, autochthonous transmission to humans has been reported in the state (Dorn *et al*., 2007).

*Trypanosoma cruzi* is a generalist parasite known to infect a great diversity of mammalian species with recent evidence also suggesting infection of certain avian species (Martínez-Hernández *et al*., 2022). Furthermore, the parasite itself is highly genetically diverse and is currently classified into 7 distinct lineages or discrete typing units (DTUs): TcI–TcVI and the more recently described TcBat (Zingales *et al*., 2009; Marcili *et al*., 2009*a*; Lima *et al*., 2013). Further complicating the picture of genetic diversity, 2 of these lineages, namely TcV and TcVI, arose from hybridization events of TcII and TcIII. There is also increasingly recognized intra-lineage diversity, with DTU TcI subdivided into TcIa–e (Herrera *et al*., 2007; Falla *et al*., 2009; Cura *et al*., 2010) and TcIV structured into distinct lineages in North and South America (Marcili *et al*., 2009*b*; Lewis *et al*., 2011; Flores-López *et al*., 2022).

Accordingly, transmission networks are complex and difficult to elucidate. While parasite diversity has been proposed to be associated with geography, particular ecology, host specificity and clinical prognosis of human infections (Llewellyn *et al*., 2009; Marcili *et al*., 2009*b*; Zingales *et al*., 2012; Messenger *et al*., 2015; Izeta-Alberdi *et al*., 2016), clear associations have been difficult to establish. Recent work in Louisiana has evidenced that several mammalian hosts are involved in shared transmission networks, including dogs, rodents and others (Dumonteil *et al*., 2020*a*, 2020*b*). Thus, a better understanding of local transmission networks is a critical piece towards accurately assessing how parasite diversity affects the risk of both infection and disease in humans.

Though early reports of *T. cruzi* genotypes circulating in the US identified only DTUs TcI and TcIV (Bern *et al*., 2011), DTUs TcII, TcV and TcVI have been identified in more recent years. Indeed, parasites from the closely related DTUs II/V/VI were identified in autochthonous human cases in Texas in a blood donor study, though exact DTU could not be determined given the sequencing approach used (Garcia *et al*., 2017). With the advent of next-generation sequencing approaches that allow for the identification of multiple genotypes per sample as well as low-frequency genotypes, the presence of DTUs TcII, TcV and TcVI was also confirmed in wild and domestic animals in Louisiana (Pronovost *et al*., 2018; Dumonteil *et al*., 2020*a*, 2020*b*, 2021).

It has been more than 60 years since *T. cruzi* was first reported in raccoons (*Procyon lotor*) in the US (Walton *et al*., 1958), and as genotyping efforts have been made throughout that time, raccoons have been described as predominantly or even almost exclusively infected with TcIV with a few known exceptions of TcI infection (Barnabé *et al*., 2001; Roellig *et al*., 2008, 2013; Bi *et al*., 2010; Bern *et al*., 2011; Curtis-Robles *et al*., 2016; Vandermark *et al*., 2018; Hodo *et al*., 2020). These genotyping studies include raccoons from Florida, Georgia, Texas, Maryland, Tennessee, Kentucky, Illinois and Louisiana. Notably, one previously characterized raccoon isolate from the Orleans Parish (NOLA) area in Louisiana was determined to be TcI (Barnabé *et al*., 2001). Though sample sizes have tended to be too small for these studies to make concrete associations, it has been repeatedly suggested that raccoons have a particular susceptibility for infection with TcIV, and to a lesser extent TcI. However, it has also been demonstrated experimentally that raccoons are competent hosts for DTU TcII as well as TcI and TcIV (Roellig *et al*., 2009*a*).

Previously, raccoons were reported as an important reservoir species for *T. cruzi* in 2 metropolitan areas of southern Louisiana, with infection prevalence reaching nearly 43% (Majeau *et al*., 2020). Beyond having one of the highest *T. cruzi* prevalence rates among mammalian hosts, raccoons also maintain a high parasitaemia for up to 5 weeks post infection (Roellig *et al*., 2009*a*; Bern *et al*., 2011). Considering the peridomestic nature of these animals and their proximity to humans, along with evidence of vectors feeding frequently on both raccoons and humans (Waleckx *et al*., 2014; Gorchakov *et al*., 2016; Dumonteil *et al*., 2020*b*), raccoons may well contribute to an increased risk of *T. cruzi* infection in humans and it is important to further elucidate the role of raccoons in local transmission cycles. Thus, *T. cruzi* genetic diversity was assessed in raccoons from southern Louisiana through deep sequencing of mini-exon amplicons derived from hearts and colons, to better understand the role of raccoons in local transmission cycles.

## Materials and methods

### Study population

Raccoons were sampled with a convenience method, including nuisance reports for raccoons in close proximity to humans and domestic animals, and tested for parasite infection with polymerase chain reaction (PCR) as described previously (Majeau *et al*., 2020). Briefly, between October 2014 and August 2018, raccoons were trapped, euthanized and necropsied from 46 trapping sites across 2 metropolitan areas in Louisiana approximately 130 km apart (Baton Rouge, BR and New Orleans, NOLA). Hearts and colons were collected and maintained at −20° until DNA extraction and PCR targeting both the satellite region and the mini-exon. Forty of the 119 raccoons collected were previously confirmed to be PCR-positive in at least 1 tissue (Majeau *et al*., 2020) and used for genotyping, 27 from NOLA and 13 from BR. All sequences used for genotyping were mini-exon amplicons.

### Amplification and sequencing

For genotyping, parasite DNA was amplified using 2 protocols targeting the mini-exon sequence (Souto *et al*., 1996; Majeau *et al*., 2019). Amplicons resulting from these 2 protocols were pooled for each sample and purified using the Invitrogen PureLink kit. Following end-repair and indexing, libraries were prepared and sequenced on a MiSeq (Illumina, Applied Biological Materials Inc. (abm) | #1-3671 Viking Way, Richmond, BC, V6V 2J5, Canada) platform.

### Sequence analysis

Sequences were analysed using Geneious Prime software. Raw Fastq reads were mapped to reference mini-exon sequences representing each of the 7 DTUs, including TcI Raccoon70 (EF576837), TcII Tu18 (AY367125.1), TcIII M6241 (AF050522), TcIV MT4167 (AF050523), TcV MN (AY367128.1), TcVI CL (U57984) and TcBat TCC949cl3Bra (KT305873). Poor quality reads and those representing less than 1% of reads were removed and sequence variants were identified for each DTU using the FreeBayes/Find SNPs plugin. Sequences were deposited into GenBank under accession numbers OP311929–OP312049.

Maximum likelihood phylogenetic trees were constructed using PHYML as implemented in phylogeny.fr including separate iterations of trees for DTU TcI only, DTUs TcII/TcV/TcVI only and DTU TcIV only. Mini-exon sequences from triatomine vectors, dogs, cats, rodents, non-human primates and humans from North America (Pronovost *et al*., 2018; Villanueva-Lizama *et al*., 2019; Herrera *et al*., 2019*a*; Dumonteil *et al*., 2020*a*, 2020*b*, 2021) were also included for comparison.

Sequence read counts for each haplotype were used to calculate the proportions of both haplotypes and DTUs for each individual raccoon tissue sample to assess parasite diversity in raccoons and between the 2 metropolitan areas. For a broader analysis across southern Louisiana, *T. cruzi* DTU cumulative proportions from triatomine vectors, dogs, cats, rodents, non-human primate hosts (Pronovost *et al*., 2018; Herrera *et al*., 2019*a*; Dumonteil *et al*., 2020*a*, 2020*b*, 2021) and racoons (this study) were calculated for 20 parishes in Louisiana for which data were available, and associated with the corresponding level III ecoregions based on U.S. Geological Survey (USGS) classification (Daigle *et al*., 2006). The following parishes were included Ascension, Calcasieu, De Soto, East Baton Rouge, Iberia, Jackson, Lafourche, Livingston, Orleans, Plaquemines, St. Bernard, St. John, St. Tammany, Tangipahoa, Vernon, Walker, Washington and West Baton Rouge. Differences in DTU proportion among ecoregions was assessed by *χ*^2^ test. A map of DTU distribution according to ecoregions was elaborated in qGIS.

Finally, phylogenetic trees were constructed to investigate parasite diversity distribution across the heart and colon within the same raccoon hosts. For each raccoon with high-quality *T. cruzi* sequences isolated from both heart and colon samples, sequences were aligned with reference sequences and maximum likelihood trees constructed, as above.

## Results

Given the importance of raccoons as a reservoir for the *T. cruzi* parasite, the full diversity of parasites infecting racoons in 2 metropolitan areas of Louisiana was identified through next-generation sequencing. From a total of 40 *T. cruzi*-positive raccoons, sequences from 29 tissue samples across 25 individual raccoons (62.5%) were successfully genotyped. Of these, 13 raccoons were from NOLA and 12 were from BR. After filtering as described above, a total of 121 haplotypes were recovered from all racoon tissue samples, 98 of which were unique to a single raccoon and 23 of which were found in multiple samples. Between 1 and 12 haplotypes were recovered per raccoon (mean = 4.84, median = 4). On average, a similar number of haplotypes were recovered per raccoon at each of the 2 sites, with a mean of 4.6 haplotypes per raccoon in BR and a mean of 4.0 haplotypes per raccoon in NOLA. Although a greater number of hearts than colons were both found to be infected and successfully genotyped, the number of haplotypes recovered from each tissue type was similar. Between 1 and 10 haplotypes were found per heart (mean = 4.5, median = 4) while between 2 and 8 haplotypes were found per colon (mean = 4, median = 4). Two raccoons from each metropolitan area (NOLA and BR) had high-quality *T. cruzi* sequences isolated from both their hearts and colons, resulting in a total of 4 raccoons with paired samples data.

**Fig. 1.**
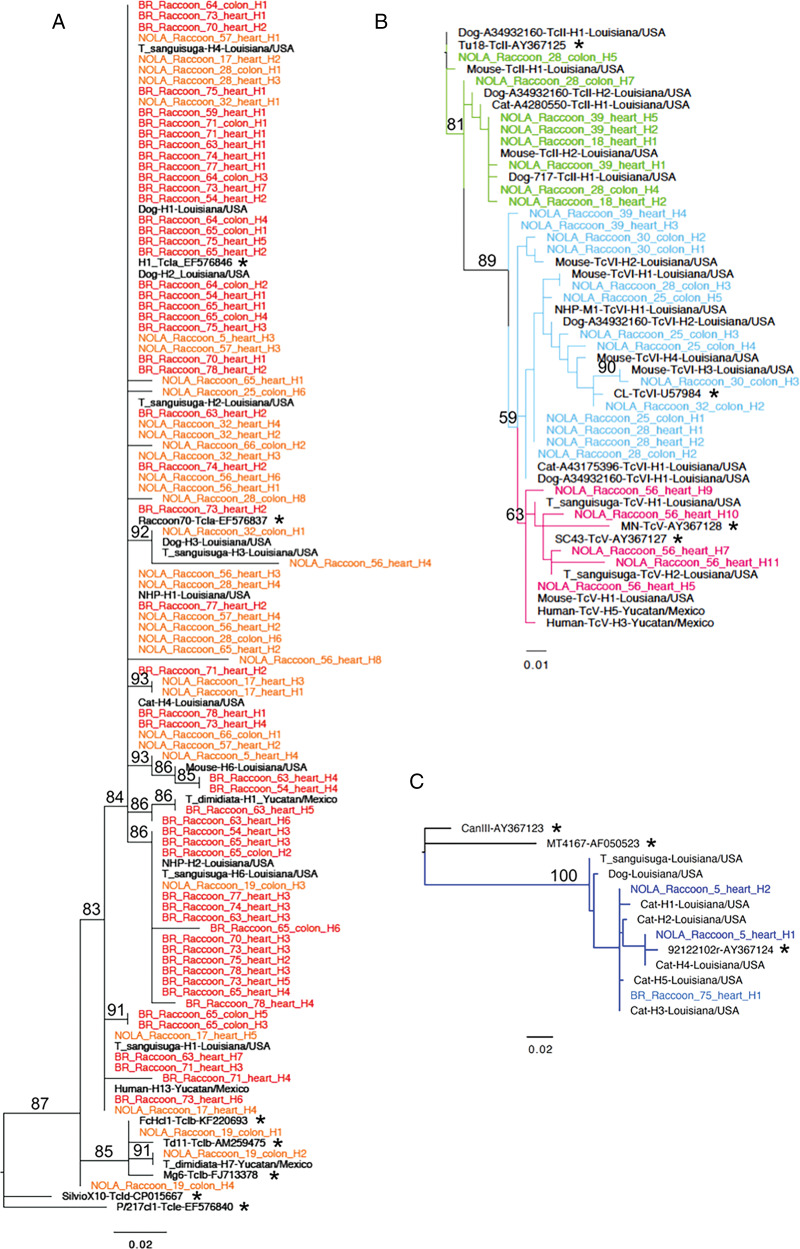
Maximum likelihood trees of *T. cruzi* mini-exon sequences. Maximum likelihood phylogenetic trees were constructed for raccoon sequences identified as TcI (A), TcII/TcV/TcVI (B) and TcIV (C), with reference sequences (asterisks) and sequences from other mammals and vectors included for each iteration (black). Most TcI sequences (A) from both NOLA (orange) and BR (red) were TcIa and closely related to other TcIa sequences from local vectors, though TcIb was also identified in NOLA. All 3 of the closely related DTUs TcII, TcV and TcVI were identified in raccoons in NOLA (B), with TcII sequences in green, TcV sequences in pink and TcVI sequences in blue. TcIV sequences (C) were identified in both NOLA (dark blue) and BR (light blue), with all sequences clustering closely with the North American TcIV sequence.

Maximum likelihood phylogeny trees were constructed using all parasite sequences identified in raccoon samples, along with reference sequences from each DTU and additional *T. cruzi* sequences previously identified in mammals and vectors from North America. A greater parasite diversity than previously reported was found to be infecting raccoons in Louisiana, with TcI, TcII, TcIV, TcV and TcVI being detected (Fig. 1), although the majority of sequences belonged to TcI DTU. Separate iterations of maximum likelihood phylogeny trees were constructed for DTU TcI (Fig. 1A), DTUs TcII/TcV/TcVI (Fig. 1B) and DTU TcIV (Fig. 1C) to better visualize intra-lineage diversity and better resolve the closely related DTUs TcII, TcV and TcVI.

Many of the TcI sequences from both NOLA and BR were closely related and clustered with TcIa reference sequences (Fig. 1A). Interestingly, these sequences also clustered with TcIa sequences derived from local triatomine vectors as well as other mammals in Louisiana, confirming that raccoons are an important part of local transmission cycles. Sequences from 1 NOLA raccoon also clustered with TcIb reference sequences and sequences from a vector in Mexico. There did not appear to be any TcIc, TcId or TcIe present in raccoons from either metropolitan area. While there appears to be some degree of clustering of TcI sequences by location between the 2 metropolitan regions, identical or very closely related sequences were also found in both NOLA and BR.

Parasites from TcII, TcV and TcVI DTUs were identified only in raccoons from NOLA but not in BR (Fig. 1B). Within each DTU, sequences from raccoons clustered closely with sequences from triatomine vectors and other mammals in Louisiana, providing further evidence of ongoing peridomestic transmission cycles in the US involving these parasite lineages, at least in the NOLA area.

The analysis of TcIV sequences (Fig. 1C) indicated that sequences from raccoons from both NOLA and BR clustered closely with other TcIV strains from the US yet distinctly from the Brazilian/South American TcIV reference strain.

**Fig. 2.**
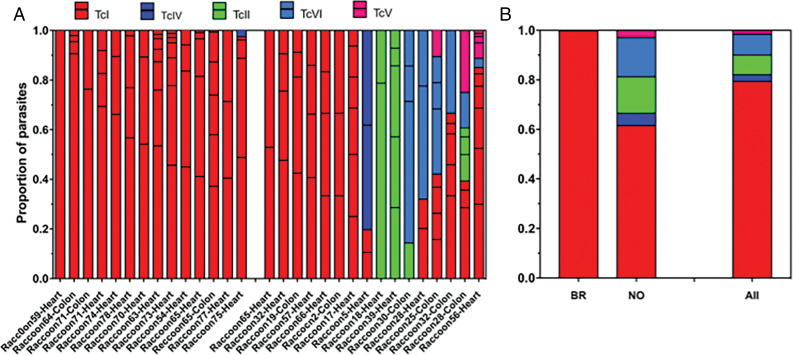
Frequency of *T. cruzi* parasite DTUs. Multiple genotypes were identified in all but 1 raccoon (A), with 10 samples demonstrating mixed infections with multiple DTUs. DTU TcI was the most abundant DTU in both metropolitan locations (NO, New Orleans and BR, Baton Rouge) as well as overall (B), followed by TcII and TcVI, TcIV and lastly TcV.

Next, the proportion of parasite haplotypes and DTUs was compared across individual raccoon samples and between regions (Fig. 2). Multiple sequence haplotypes were found in all but one of the samples tested (Fig. 2A). Interestingly, 13/14 samples from 12 raccoons from the BR metropolitan area harboured exclusively TcI parasites and only 1 raccoon presented a co-infection with TcI and TcIV, although TcI predominated. On the other hand, in NOLA metropolitan area, 7/16 raccoons harboured only TcI parasites, 2/16 raccoons were infected with only TcII and 7/16 raccoons presented mixtures of TcI, TcII, TcIV, TcV and TcVI in different proportions (Fig. 2A), with up to 4 DTUs detected in a single raccoon. Despite TcIV being reported as the predominant DTU present in raccoons in the US (Hodo and Hamer, 2017; Hodo *et al*., 2020), it was only detected in 2 raccoons, 1 in each metropolitan area of Louisiana. When comparing *T. cruzi* DTU distribution in raccoons from the 2 regions, a striking difference was observed with racoons in NOLA infected with a large diversity of DTUs, with TcII, TcIV, TcV and TcVI accounting for 38.5% of haplotypes, while TcI largely predominated in BR, representing 99.8% of haplotypes (Fig. 2B). DTU TcIV was found at a low frequency, comprising only around 3% of all sequences identified. Overall, DTU TcI was the most abundant DTU identified, comprising nearly 80% of all sequences (Fig. 2B) and all but 3 raccoons harboured parasites from DTU TcI, often as a mixed infection with other DTUs in raccoons from the NOLA region.

**Fig. 3.**
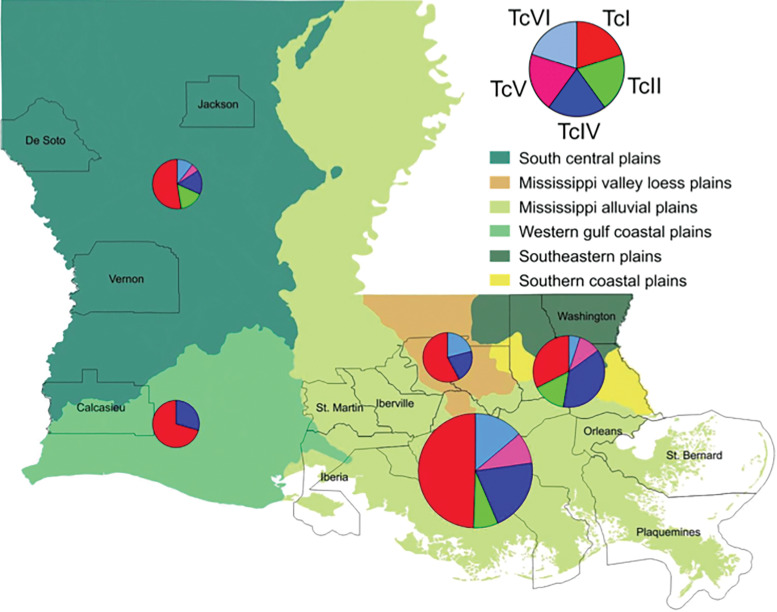
Map of *T. cruzi* DTU distribution in Louisiana ecoregions. Cumulative proportions of DTUs from multiple mammal and vector samples across 21 parishes in Louisiana were mapped to level III ecoregions. Parishes from which *T. cruzi* DTU data were available from vectors/hosts are outlined. Distribution of DTUs varies significantly across level III ecosystems in Louisiana (*χ*^2^ = 30.6, d.f. = 16). Size of each pie chart circle indicates the total number of sequences represented.

These data suggest that raccoons may not present major differences in susceptibility to *T. cruzi* DTUs. Rather, differences in *T. cruzi* DTU distribution in raccoons between BR and NOLA raccoons may reflect geographical/ecological differences. To test this, we performed a broader comparison of *T. cruzi* DTU distribution across southern Louisiana based on available data from multiple hosts and vectors. As expected, *T. cruzi* DTU distribution in Louisiana was found to vary significantly according to level III ecoregions (Fig. 3, *χ*^2^ = 30.6, d.f. = 16, *P* = 0.015), suggesting that the observed differences in DTU proportion in raccoons between BR and NOLA likely reflect ecological differences in parasite diversity. Accordingly, Mississippi valley loess plains (including the BR area) and Western gulf coastal plains appear to support a lower diversity of *T. cruzi* DTUs compared to other ecoregions in Louisiana.

**Fig. 4.**
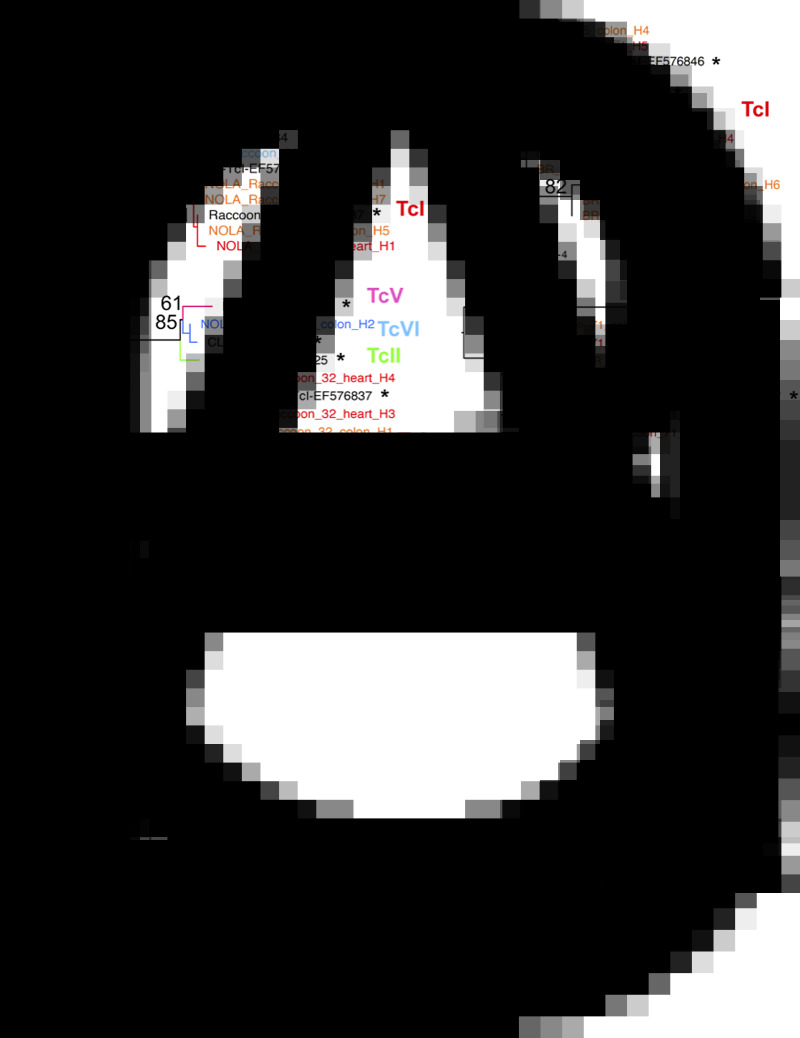
*Trypanosoma cruzi* haplotype distribution across tissues within individual raccoons. Two raccoons in each metropolitan area had high-quality *T. cruzi* sequences isolated from both hearts and colons. The 2 NOLA raccoons (A and C) each had infection with multiple DTUs, while the 2 BR raccoons (B and D) were infected with TcI. In each of the 2 NOLA raccoons (A and C), some DTUs were found only in the colon. Within DTU TcI in all 4 panels, some haplotypes were found in both tissues tested, while others were only detected in the heart or the colon.

Finally, to investigate *T. cruzi* haplotype distribution across tissues within the same raccoon hosts, phylogenetic trees were constructed for sequences from 4 individual raccoons that had paired heart and colon samples in which *T. cruzi* was successfully genotyped. While some DTUs or haplotypes were found in both the heart and the colon of a single individual, others appeared to have tropism for a particular tissue (Fig. 4). In NOLA Raccoon 28 (Fig. 4A), identical or very closely related TcI and TcVI sequences were found in both the heart and the colon, but TcII sequences were only found in the colon. Similarly, in NOLA Raccoon 32 (Fig. 4C), some TcI sequences were found in both tissues, but a particular cluster of sequences was found only in the heart. Furthermore, TcVI was found only in the colon of this raccoon. While only TcI was identified in BR Raccoon 65 (Fig. 4B), some haplotypes were found in both the heart and the colon, but others were found in only 1 of the 2 tissues. Finally, in BR Raccoon 71, no TcI haplotype was found to be unique to the colon, but 1 cluster appeared to be unique to the heart (Fig. 4D).

## Discussion

While raccoons have been long reported as an important reservoir due to their high and persistent parasitaemia as well as their proximity to humans, insights into the genetic diversity of *T. cruzi* circulating in this species further confirm their importance in transmission cycles in Louisiana and ultimately in relation to human health risk. The presence of highly similar or identical sequences to those found in other mammalian hosts and vectors in the region confirms the previously hypothesized role of raccoons in these shared local transmission networks. Though the circulation of DTUs TcII, TcV and TcVI in Louisiana has previously been confirmed in several mammalian hosts, with DTUs TcII and TcV also having been identified in local *T. sanguisuga* vectors, this is the first report of these DTUs also infecting raccoons in the US.

While it has been previously suggested that raccoons may be more susceptible to TcIV infection, and/or were preferentially involved in parasite transmission cycles associated with this DTU (and to a lesser extent TcI), the observation of natural infection with multiple other DTUs using a deep sequencing approach rather refutes this hypothesis. Indeed, based on our data and previous reports, racoons appear to be equally susceptible to infections with a large diversity of *T. cruzi* strains covering TcI, TcII, TcIV, TcV and TcVI DTUs. Thus, while differences in host susceptibility to *T. cruzi* DTUs cannot be ruled out for other mammalian species, ecology is emerging as an important driver of DTU circulation and distribution. Distinct transmission networks may be the result of the interactions among unique host assemblages and vector feeding profiles in each habitat, together with the infection characteristics in each host species. The lack of observed TcII, TcV and TcVI DTUs in raccoons from the BR metropolitan area suggests independent local transmission cycles compared to the NOLA area, shaped by larger ecological differences and allowing for a greater diversity of parasite DTUs in NOLA. Accordingly, further targeted sampling of mammals using deep sequencing approaches is key to fully understand parasite transmission networks in the different habitats and ecoregions and ultimately threats to human health.

Considering the difference observed in proportions of DTUs as well as individual haplotypes within DTU, it becomes clear that deep sequencing methods are essential for assessing *T. cruzi* diversity in biological specimens. Though the mini-exon marker is polymorphic and cannot be used to determine whether multiple sequence types from the same DTU represent multiclonal infection or gene diversity within a monoclonal infection, nearly half the raccoons from NOLA and 1 raccoon from BR harboured parasites from multiple DTUs, providing evidence of multiclonal infection. It has been nearly 20 years since the first evidence that *T. cruzi* genetic exchange can occur within vertebrate hosts when infected with multiple lineages (Gaunt *et al*., 2003), with recent literature emphasizing that this may occur *via* multiple mechanisms (Schwabl *et al*., 2019). Infection with multiple genotypes in the same host, therefore, provides opportunity for the generation of greater parasite diversity through recombination events. Thus, while Sanger sequencing and other less sensitive genotyping methods continue to be used in *T. cruzi* genotyping studies, deep sequencing approaches may be truly necessary to elucidate all lineages present in an infection and more fully understand *T. cruzi* transmission cycles, infection dynamics and fundamentally risk to humans across the Southern US and elsewhere.

As exact associations between specific genotypes and clinical disease outcomes continue to be investigated and as the full extent of intra-lineage diversity is further elucidated, it is important to consider associations at both the DTU level and at the specific haplotype level. Indeed, while some previous studies report DTU associations with specific outcomes, others fail to find such associations. Tissue tropism of various genotype groupings is, then, potentially important to consider as a contributing factor to clinical outcomes. Though a very limited dataset, it is interesting that TcII was only found to be present in the colon of NOLA Raccoon 28 and not in the heart, as TcII has been associated with digestive complications of Chagas disease (Nielebock *et al*., 2020). Looking at TcI, however, while there is no apparent DTU-level tropism, there does seem to be specific haplotype-level differences in tropism, which may, in part explain some of the clinical variation seen in infections with this DTU. Differences in clinical outcomes between genotypes with specific tissue tropisms *vs* those that seem to infect the host more broadly are certainly interesting to explore, but a greater depth of information remains crucial in teasing out these nuanced details, especially when considering multiclonality of infection.

Fundamentally, One Health approaches to anthropozoonoses are necessary to best estimate and mitigate risk to human health; the results presented herein support the need for widespread monitoring of *T. cruzi* in wildlife across the US. DTU TcVI has been described as presenting a risk for both the cardiac and the gastrointestinal forms of the disease and yet was thought to be essentially absent in sylvatic cycles from South America (Bizai *et al*., 2020; Monje-Rumi *et al*., 2020; Nielebock *et al*., 2020). If this DTU is found to be circulating more in wildlife than previously expected, there may be an increased risk to local humans for clinical forms of Chagas disease. Similarly, the presence of TcII haplotypes with a seeming tropism for the colon in the NOLA area transmission cycle suggests that these genotypes could cause gastrointestinal complications in an autochthonous human infection. Furthermore, TcIa, 1 of the 2 subtypes of TcI identified in these raccoons, has been associated with severe heart disease and reactivation of disease after transplant in Argentina (Burgos *et al*., 2010; Herrera *et al*., 2019*b*). These findings are particularly concerning when paired with repeated evidence of triatomine vectors feeding on both raccoons and humans.

In conclusion, raccoons in the US are confirmed as an important reservoir species for *T. cruzi* and harbour a greater diversity of parasite than previously identified. Geographic differences in parasite diversity infecting these raccoons argue against host-specific differences in susceptibility to *T. cruzi* DTUs, and rather suggest that ecological niches play a significant role in shaping the distribution of parasite diversity, highlighting the existence of punctuated, local transmission cycles. Given both this finding and the evidence that some haplotypes may have tissue-specific tropisms, which may contribute to differences in infection outcomes, widespread *T. cruzi* surveillance in reservoir species with next-generation sequencing approaches remains an important component of assessing risk to human health in the US.

## Data Availability

Data are available on NCBI under accession numbers OP311929–OP312049.
